# Effects of Radiofrequency Electromagnetic Radiation on Neurotransmitters in the Brain

**DOI:** 10.3389/fpubh.2021.691880

**Published:** 2021-08-17

**Authors:** Cuicui Hu, Hongyan Zuo, Yang Li

**Affiliations:** ^1^Anhui Medical University, Academy of Life Sciences, Hefei, China; ^2^Department of Experimental Pathology, Beijing Institute of Radiation Medicine, Beijing, China

**Keywords:** EMR, brain, neurotransmitter, metabolism, transmission, receptor

## Abstract

With the rapid development of electronic information in the past 30 years, technical achievements based on electromagnetism have been widely used in various fields pertaining to human production and life. Consequently, electromagnetic radiation (EMR) has become a substantial new pollution source in modern civilization. The biological effects of EMR have attracted considerable attention worldwide. The possible interaction of EMR with human organs, especially the brain, is currently where the most attention is focused. Many studies have shown that the nervous system is an important target organ system sensitive to EMR. In recent years, an increasing number of studies have focused on the neurobiological effects of EMR, including the metabolism and transport of neurotransmitters. As messengers of synaptic transmission, neurotransmitters play critical roles in cognitive and emotional behavior. Here, the effects of EMR on the metabolism and receptors of neurotransmitters in the brain are summarized.

## Background

Electromagnetic radiation (EMR) is closely related to human life and originates from various electrical systems, such as mobile phones, microwave ovens, communication base stations, high-voltage lines, electronic instruments and other electromagnetic equipment. EMR produces various electromagnetic waves of different frequencies, resulting in the increasing EMR intensity in human living spaces. The high-frequency waves such as cosmic, gamma and X-rays, have enough energy to cause ionization. Non-ionizing electromagnetic waves, including ultraviolet, visible region, infrared, microwave, and radio waves are frequently used in daily life, especially radiofrequency electromagnetic fields (RF-EMFs, 30 kHz-300 GHz) for communications, and extremely low-frequency EMFs (ELF-EMFs, 3 Hz-3 kHz) generated by electricity. RF is also commonly referred to as microwave (MW) radiation. The impact of EMR on human health has also gradually attracted attention, and the modulation of brain functional connectivity was observed in human body (1–3). This review summarizes the effects of RF-EMF on neurotransmitters in the brain.

The effects of EMR on body systems might depend on the frequency, intensity and power of radiation, so the parameters of EMR provide a challenge for a literature review. Specific absorption rate (SAR) measures the rate of energy absorbed by the human body when exposed to electromagnetic fields between 100 kHz and 10 GHz. With the unit of watt per kilogram (W/kg), SAR reflects the power absorbed per mass of tissue. The SAR value depends on the frequency, incident direction, E-polarization direction, and the structure of different tissues.So far, the SAR values range from 10^−4^ to 35 W/kg in those reported studies on the bioeffects of microwave radiation. Numerous studies have shown that the nervous system is an important target organ system sensitive to EMR. Exposure to electromagnetic fields can cause structural and functional changes in the nervous system (4–7). Neurotransmitters are specific chemicals that act as messengers during synaptic transmission within the nervous system. Many studies have shown that EMR affects the metabolism and transport of neurotransmitters (8). It is well understood that neural circuit is the structural basis of brain function, and the brain works by the interplay of various brain regions and many neurotransmitters. Consequently, the modulatory effect of EMR on neurotransmitter levels in various brain regions may play a critical role in the brain functioning. According to many studies, RF-EMR exposure can induce the imbalance of amino acid neurotransmitters in various parts of the brain (9, 10).

Neurotransmitters are synthesized by nerve cells and transported into the synaptic vesicles of presynaptic cells. Through action potentials, transmitter release at synaptic endings is mediated by calcium ion channels; transmitters are then diffused through the synaptic cleft and act on specific receptors on postsynaptic neurons or effector cells, thus transferring information from presynapses to postsynapses (11). The action of neurotransmitters can be discontinued by recycling; that is, excess neurotransmitters in the synaptic cleft are recycled to presynaptic neurons by the action of presynaptic vectors and are stored in vesicles. Neurotransmitter activity can also be aborted by enzymatic hydrolysis; for instance, dopamine (DA), is metabolically inactivated by the actions of monoamine oxidase located in mitochondria and catechol-O-methyltransferase (COMT) located in the cytoplasm (12). Neurotransmitters are involved in the processes of brain development, including neurotransmission, differentiation, and the formation of neural circuitry. They enable neurons to communicate with each other, and alterations in the levels of specific neurotransmitters are related to various neurological disorders, such as depression, schizophrenia, Alzheimer disease, and Parkinson disease (13). Neurotransmitters in the central nervous system are usually divided into four categories based on their chemical constitution. Biogenic amines include DA, norepinephrine (NE), epinephrine (E), 5-hydroxytryptamine (5-HT), etc. Amino acids include γ-aminobutyric acid (GABA), glycine, glutamate, acetylcholine (Ach), etc. Peptide neurotransmitters include endogenous opioid peptides and other varieties. The remaining category of transmitters includes other types, such as nitric oxide (NO) and substance P. Pertinently, the current review discusses the pivotal studies that shed light on the neurotransmitters in the brain in the above mentioned four categories when they encountered EMR exposure, thus providing an overview of the metabolism and receptor changes of these neurotransmitters.

For the literature retrieval, we searched all the articles in the NCBI PubMed database, with the keywords of each “neurotransmitter” and “electromagnetic field” or “radiofrequency electromagnetic field,” and selected the published articles written in English and referring to neurotransmitter measurement in the brain encounter with RF-EMF exposure. Overall, 21 articles related to neurotransmitters with short-term EMR exposure were discussed in the text and summarized in Table 1, and 19 articles related to neurotransmitters with long-term EMR exposure were discussed in the text and summarized in Table 2.

**Table 1:** The influence of short-term EMR exposure on neurotransmitters in the brain.

**Neurotransmitter**	**Reference**	**Sample/Model**	**Exposure condition**	**Results**
DA	Aboul Ezz et al. (14)	Adult rats	1800 MHz, 0.843 W/kg, 0.02 mW/cm^2^, 1 h	DA decrease in the hippocampus
	Inaba et al. (15)	Rats	2450 MHz, 5 and 10 mW/cm^2^, 1 h	DOPAC increase in the pons and medulla oblongata at 10 mW/cm^2^
5-HT	Inaba et al. (15)	Rats	2450 MHz, 5 and 10 mW/cm^2^, 1 h	5-HIAA increase in the cerebral cortex at 5 and 10 mW/cm^2^. 5-HIAA:5-HT ratio increase in the cerebral cortex at 5 mW/cm^2^. 5-HT turnover rate increase in the pons and medulla oblongata
	Ishikawa et al. (16)	Male Wistar rats	2450 MHz, 5 kW; 0.5 or 1.5 s	5-HIAA decrease at 0.5 s; 5-HIAA increase at 1.5 s
Glutamate & aspartic acid	Karri et al. (17)	Wistar rats	30 mW/cm^2^, 10 min	Glutamate and aspartic acid decrease 1 day after radiation
	Wei et al. (1)	Male Wistar rats	5,10,30, and 100 mW/cm^2^; 5 min	Glutamate and aspartic acid decrease in the hippocampus within 7 days after radiation, especially in the 10 mW/cm^2^ group
	Mausset-Bonnefont et al. (18)	Rats	900 MHz, 6 W/kg, 15 min	NR1 decrease in the cortex; NR2A decrease in the cortex and hippocampus; NR2B decrease in the striatum
	Zhang et al. (19)	Rats	12.0 W/kg, 65 mW/cm^2^, 20 min	NR1 decrease at 3 h, 24 h, and 3 days; NR2A decrease at 0 h, 3 h, and 12 h; NR2C decrease at 0 h and 24 h; NR2D increase at 0 h, 12 h, 24 h, and 3 days in the hippocampus after radiation
	Wang et al. (20)	Rats	65 mW/cm^2^, 12.0 W/kg, 20 min	NR1, NR2A, NR2C decrease; NR2D increased in the hippocampus
	Xiong et al. (21)	Male Wistar rats	2.856 GHz, 30 mW/cm^2^, for 10 min every other day three times	NR2A increase at 7 days; NR2B increase at 1 day in the hippocampus
GABA	Qiao et al. (22)	Wistar rats	30 mW/cm^2^, 5 min	GABA decrease in the hippocampus
	Wang et al. (23)	Wistar rats	2.856 GHz, 50 mW/cm^2^, 6 min	GABA decrease at 6 months
	Noor et al. (9)	Male albino rats	900 MHz, 1.165 W/kg, 0.02 mW/cm^2^, 1 h/day	Glycine increase in the midbrain after 1 month
	Wang et al. (24)	Rats	900 MHz, 2.23 W/kg, 6 mW/cm^2^	GABA receptor upregulation in cultured neurons
ACh	Fujiwara et al. (25)	Mice	2.45 GHz	Transient elevated ACh content in the brain
	Lai et al. (26)	Rats	2.45 GHz, 0.6 W/kg, 20 min	Choline uptake activity increase in the frontal cortex, hippocampus, and hypothalamus
	Krylova et al. (27)	Rats	2.35 GHz, 1 mW/cm^2^	Decreased mAChR activity; increased number of mAChR receptors in the cerebral cortex
	Testylier et al. (28)	Rats	2.45 GHz, 4 mW/cm^2^, 1 h	ACh decrease in the hippocampal CA1 area
	Lai et al. (29)	Rats	Pulsed (2 μs, 500 pps) or continuous wave 2450 MHz microwaves, 45 min	Choline uptake decreased in the frontal cortex
Peptides	Lai et al. (30)	Rats	2450 MHz, 0.6 W/kg, 1 mW/cm^2^, 45 min	Three subtypes of opioid receptor blocked cholinergic activity decrease in the hippocampus induced by microwave radiation
	Lai et al. (31)	Rats	2450 MHz, 0.6 W/kg, 1 mW/cm^2^, 45 min	Pretreatment with opioid antagonists naltrexone or cholinergic agonists inhibited microwave-induced radial arm maze learning disorders
NO	Burlaka et al. (32)	Wistar rats	0.465 GHz, 1.0-6.0 mW/cm^2^ pulse duration 2 ms, 17.5 min	NO synthesis increase in mitochondria of neural cells, NO synthase increase in the brain

**Table 2:** The influence of long-term EMR exposure on neurotransmitters in the brain.

**Neurotransmitter**	**Reference**	**Sample/Model**	**Exposure condition**	**Results**
DA	Kim et al. (33)	C57BL/6 mice	835 MHz, 4.0 W/kg, 5 h/day for 12 weeks	DA decrease in the striatum
	Maaroufi et al. (34)	Rats	900 MHz, 1 h/day for 21 days, 0.05 W/kg < SAR < 0.18 W/kg	DA decrease in in the hippocampus
	Ji et al. (35)	Pregnant Wistar rats	900 MHz, 3 times daily for 20 days; 10, 30, or 60 min each time	DA increases in brain tissue of both pregnant and fetal mice in the 10 min group; DA decrease in the 60 min group
NE and E	Megha et al. (36)	Male Fischer 344 rats	1800 MHz, 1 mW/cm^2^, 2 h/day, 5 days/week for 30 days	NE and E decrease in the hippocampus
	Cao et al. (37)	Male LACA mice	900 MHz, 0, 1, 2 and 5 mW/cm^2^; 0, 0.22, 0.44 or 1.1 W/kg; 1 h/day for 35 days	NE increase at 1 mW/cm^2^
	Ji et al. (35)	Pregnant rats	900 MHz,0.9W/kg, m 3 times daily for 20 days; 10, 30, or 60 min each time	NE increase in the 10 min group; NE decrease in the 60 min group
5-HT	Li et al. (38)	Wistar rats	2.856 GHz, 5, 10, 20, 30 mW/cm^2^; 3 times per week for up to 6 weeks	5-HT increase in the hippocampus from 28 days to 2 months at 30 mW/cm^2^
	Aboul Ezz et al. (14)	Adult rats	1800 MHz, 1 h/day for 1, 2 and 4 months, 0.843 W/kg,0.02 mW/cm^2^	5-HT increase in the hippocampus, hypothalamus and midbrain
	Maaroufi et al. (34)	rats	900 MHz, 1 h/day for 21 days, 0.05 W/kg < SAR < 0.18 W/kg	5-HIAA decrease, the 5-HIAA/5-HT ratio decrease in the cerebellum
Glutamate & aspartic acid	Ahmed et al. (39)	Rats	1800 MHz, 0.843 W/kg, 0.02 mW/cm^2^; 1 h/day for 1, 2, or 4 months	Glutamate and glutamine decrease in the hippocampus after 1 month
	Wang et al. (20)	Wistar rats	30 mW/cm^2^ for 5 min/day, 5 days/week, 2 months	Glutamate increase and NR2B decrease in the hippocampus and cerebrospinal fluid
	Zhao et al. (40)	Male Wistar rats	2.5, 5 and 10 mW/cm^2^, 6 min/day, 1 months	Glutamate and aspartic acid, increase in the 2.5 and 5 mW/cm^2^ groups and decrease in the 10 mW/cm^2^ group in the hippocampus
	Wang et al. (41)	Male Wistar rats	2.856 GHz; 0, 2.5, 5 and 10 mW/cm^2^ for 5 min/day, 5 days/week and up to 6 weeks	NR2B and p-NR2B decrease in the 10 mW/cm^2^ group; NR2A decrease in the 10 mW/cm^2^ group at 1 day and 6 months
	Huang et al. (42)	Female Wistar rats	1800 MHz, 0.5 mW/cm^2^ and 1.0 mW/cm^2^, 12 h/day for 21 days	For the 0.5 mW/cm^2^ group, NR2A decrease in the CA3 region, NR2B decrease in the CA1 and CA3 regions. For the 1.0 mW/cm^2^ group, NR2A decrease in the CA1 and CA3 regions and an NR2B decrease in the CA1 region, CA3 region, and DG
GABA	Zhang et al. (43)	Male mice	1.8 GHz, 4 weeks	GABA and aspartic acid decrease in the cortex and hippocampus
ACh	Kumar et al. (44)	Mice	2.45 GHz, 2 h/day, 1 months	M1 AChRs upregulated; AChE activity increased in the hippocampus
	Gökçek-Saraç et al. (45)	Wistar rats	2.1 GHz, 45 V/m and 65 V/m, 1 week	AChE, ChAT, and VAChT decrease in rat hippocampus after exposure to 65 V/m
	Gupta et al. (46)	Rats	2.45 GHz, 1 h/day for 28 consecutive days	ACh decrease, AChE activity increasedin the hippocampus
	Kunjilwar and Behari (47)	Rats	147 MHz, subharmonics of 73.5 MHz and amplitude of 36.75 MHz modulated at 16 Hz and 76 Hz, 3 h/day, for 30-35 consecutive days	AChE decrease in the brain
NO	Qin et al. (48)	Mice	0.9 V/m, 6 h/day, 12 h/day, and 18 h/day, 30 days	NO increase in the brain

## Effects of EMR on Biological Amine Neurotransmitters

### Effects of EMR on Dopamine (DA)

As a precursor of norepinephrine, DA is a key neurotransmitter in the hypothalamus and pituitary gland. It is mainly responsible for activity in the brain associated with reward, learning, emotion, motor control, and executive functions. DA also correlates to psychiatric and neurological disorders, including Parkinson disease, multiple sclerosis, and Huntington disease (13). It has been suggested that DA inhibits the secretion of gonadotropin-releasing hormone, and there is an axonal connection and interaction between gonadotropin-releasing hormone and DA in nerve endings (49). Deficiency of DA in the basal ganglia is seen in patients with Parkinsonism (50). DA also has some role in Schizophrenia—striatal DA is increased, and cortical DA transmission is altered (51, 52).

Several studies reported the effects of EMR on DA. For example, adult rats undergoing daily EMR exposure for 1 h, with an EMR frequency of 1,800 MHz, a specific absorption rate (SAR) value of 0.843 W/kg, power density of 0.02 mW/cm^2^, induced a significant decrease in DA in the hippocampus after 2 months of exposure and 1 month after cessation of exposure. This study indicated that EMR exposure may reduce DA production in the hippocampus, affect rat arousal, and contribute to decreased learning and memory ability after exposure to EMR (14). Maaroufi et al. exposed rats to 900 MHz EMF, 1 h/day during 21 consecutive days, with the minimum SAR 0.05 W/kg and the maximum SAR 0.18 W/kg, depending on the position of the rat in the field. A decrease in DA was observed in the hippocampus of the EMR exposed group. Moreover, there is a significant difference of DA and dihydroxyphenyl acetic acid (DOPAC) between hippocampus and striatum in the EMR exposed group (34). Furthermore, exposure to a RF-EMR of 835 MHz, SAR value of 4.0 W/kg, for 5 h/day for 12 weeks, led to a reduction in DA concentration in the striatum of C57BL/6 mice (33). The above studies suggest that a certain intensity of microwave radiation can lead to abnormal metabolism of monoamine neurotransmitters in the hippocampus and striatum.

Inaba et al. exposed adult rats to microwave radiation for 1 h, with the frequency of 2,450 MHz, and at power densities of 5 and 10 mW/cm^2^ respectively. The DOPAC content in the pons and medulla oblongata, the DA turnover rates and the DOPAC:DA ratio increased significantly in the striatum and cerebral cortex only at a power density of 10 mW/cm^2^, but no significance was observed in the DA content of any region of the brain at a power density of 5 mW/cm^2^ (15). In addition, 32 pregnant Wistar rats were divided into control group, low-dose group (receiving mobile phone radiation for 10-min periods), middle-dose group (receiving mobile phone radiation for 30-min periods), and high-dose group (receiving mobile phone radiation for 60-min periods). Rats underwent periods of radiation three times daily from the day of pregnancy continuously for 20 days. Then, the effects of mobile phone radiation on monoamine neurotransmitters in the brain tissue of fetal mice were studied, with a center frequency of 900 MHz, and a SAR value of 0.9 W/kg. The results showed that the DA content in the brain tissue of fetal mice increased in the low-dose group but decreased in the high-dose group, and no significant changes were observed in the middle-dose group, which suggested that long-term mobile phone radiation could cause abnormal DA content in the central nervous system in fetal mice and might affect the brain development of mice (35). In summary, these studies indicate that EMR can lead to metabolic disorders of monoamine neurotransmitters in the brain, depending on the intensity of radiation exposure, and might in theory result in abnormal emotional behavior.

### Effects of EMR on Norepinephrine and Epinephrine

As a neurotransmitter, norepinephrine is mainly synthesized and secreted by sympathetic postganglionic neurons and adrenergic nerve endings in the brain. A small number of norepinephrine is produced in adrenal medulla as a hormone (53). It can bind to two types of adrenergic receptors, α and β, but it mainly binds to α receptors (including α1 and α2). Norepinephrine can be converted to epinephrine through N-methylation (54). The release of norepinephrine in the brain plays a role in various processes, such as stress, attention, sleep, inflammation, and the responses of the autonomic nervous system (13). Megha et al. found that after 30 days (2 h/day, 5 days/week) of continuous 1,800 MHz, 1 mW/cm^2^ microwave radiation, the levels of norepinephrine and epinephrine in rat hippocampal tissue were significantly decreased, indicating that certain conditions of microwave radiation could lead to a decrease in norepinephrine and epinephrine contents in the brain (36). Cao et al. applied 900 MHz microwave radiation to male LACA mice. The radiation intensity used was 0, 1, 2, and 5 mW/cm^2^; the SAR values were 0, 0.22, 0.44, and 1.1 W/kg, respectively; mice were exposed for 1 h/day for 35 consecutive days. The results showed that the brain norepinephrine content increased significantly when EMR intensity was 1 mW/cm^2^, but no obvious changes in norepinephrine content were observed when exposure intensity was 2 or 5 mW/cm^2^ (37). This further suggests that low-intensity EMR exposure can cause an increase in norepinephrine content in the brain, which might in theory affect epinephrine content, leading to neurotransmitter production disorders.

Moreover, Ji et al. performed experiments on pregnant rats by exposing them to microwave radiation from 900 MHz cellular phones with the SAR value of 0.9W/kg. The control, low, middle and high dose group received 0, 10, 30, and 60 min radiation each time respectively. The radiation was applied three times a day from the first day of pregnancy for 20 consecutive days. The results showed that the norepinephrine content in the fetal rats of the low dose group increased, and the norepinephrine content in the fetal rats of the high dose group decreased significantly, compared with that in the control group (35). Together, these results suggest that long-term exposure to EMR may lead to abnormal norepinephrine and epinephrine contents in the brain, depending on the dose of radiation.

### Effects of EMR on 5-Hydroxytryptamine “Serotonin”

5-hydroxytryptamine (5-HT) is massively synthesized in the gastrointestinal tract (mainly in enterochromafin cells), whereas only a small percentage is produced within the nervous system. In the brain, 5-HT cell bodies, mainly localized in the raphe nuclei, and send axons to almost every brain region (55). As an inhibitory neurotransmitter, 5-HT is mainly distributed in the pineal gland and hypothalamus, especially in the cerebral cortex and neural synapses. 5-HT contributes to the regulation of physiological functions such as mood, feeding, cognition, memory, pain, sleep, and body temperature maintenance (56), and these physiological functions have been reported as indicators of brain injury induced by electromagnetic radiation (57). Consequently, 5-HT might play an important role in the neurobiological effects of EMR. Few studies have reported the effect of microwave radiation on 5-HT. It was reported that rats were exposed to microwave radiation for 1 h, with a frequency of 2,450 MHz, at power densities of 5 and 10 mW/cm^2^. The 5-hydroxyindoleacetic acid (5-HIAA) content in the cerebral cortex was significantly increased after microwave exposure at power densities of 5 and 10 mW/cm^2^. The 5-HT turnover rates and the 5-HIAA:5-HT ratio in the cerebral cortex increased significantly at a power density of 5 mW/cm^2^. However, there were no obvious changes in 5-HT content in the brain of microwave-exposed rats. Consistently, the 5-HT turnover rate was significantly increased in the pons, medulla oblongata and hypothalamus at a power density of 10 mW/cm^2^ (15).

Li et al. exposed Wistar rats to 2.856 GHz microwave radiation, with mean power densities of 5, 10, 20, and 30 mW/cm^2^, separately, three times per week for up to 6 weeks. Spatial learning and memory function, the hippocampal morphological structure, electroencephalogram (EEG) data and neurotransmitter content of rats were tested after the last exposure. The results showed that the content of 5-HT in the hippocampus and cerebrospinal fluid of rats in each radiation group increased significantly from 28 days to 2 months after exposure, and these changes were related to the decrease in learning and memory ability, abnormal hippocampal morphology and abnormal EEG results induced by microwave radiation (38). Maaroufi et al. reported the 5-HT increase, the 5-HIAA decrease and the 5-HIAA/5-HT ratio decrease in the cerebellum of rats, exposed to 900 MHz EMF, 1 h/day for 21 consecutive days, with the minimum SAR 0.05 W/kg and the maximum SAR 0.18 W/kg (34). Moreover, the 5-HT increase was found in the hippocampus, hypothalamus and midbrain of adult rats, after 1,800 MHz, 1 h/day for 1, 2, and 4 months EMR exposure respectively, with SAR value of 0.843 W/kg, and power density of 0.02 mW/cm^2^ (14). These studies suggest that long-term exposure to microwave radiation can lead to an increase in 5-HT in the brain, indicating a disorder in the metabolism of the neurotransmitter.

Additionally, the effect of microwave radiation on monoamine metabolism was investigated in the cortex, striatum and hippocampus of the rat brain, with the maximum power level of 5 kW at 2,450 MHz, and the radiation durations of 0.5 and 1.5 s. High-performance liquid chromatography (HPLC) with electrochemical detection was used to determine the concentrations of intracerebral monoamines and their metabolites. The concentrations of norepinephrine, DA and 5-HIAA were reduced by 0.5 s radiation. While the levels of these monoamines were increased by 1.5 s radiation (16). Whereas, another study on pregnant rats exposing to 900 MHz cellular phones, showed no significant difference in the content of 5-HT of fetal rats, in different intensities of microwave radiation groups (35). Altogether, further studies are necessary to illuminate the role of 5-HT in EMR-induced learning and memory dysfunction and morphological changes in the brain.

## Effects of EMR on Amino Acid Neurotransmitters

### Effects of EMR on Excitatory Amino Acid Neurotransmitters

Glutamate is the major excitatory neurotransmitter in the nervous system. Glutamate receptors distribute in neurons and glia of the brain and spinal cord. The C-terminus and carbon backbone of glutamate derive from glucose. After crossing the blood-brain barrier through astrocytic end feet, glucose is broken down to pyruvic acid *via* glycolysis in the cytosol. Then pyruvic acid enters the tricarboxylic acid (TCA) cycle, and α-ketoglutarate is generated. Pyruvic acid is finally transmitted to receive an amino group donated by leucine, isoleucine and valine, aspartate, γ-aminobutyric acid (GABA) and alanine etc. (58). In addition, glutamate also acts as a metabolic precursor to GABA and a component of various amino acid-based derivatives, such as the antioxidant glutathione. Metabolic studies have shown that all of the glucose is eventually converted to glutamate in the CNS, which indicating the key role of glutamate in multiple aspects of brain physiology (59, 60).

In addition to glutamate, aspartate is another excitatory neurotransmitter with high concentrations in the CNS. The synthetic and metabolic enzymes for both glutamate and aspartate are localized to neurons and glial cells, especially in the mitochondria of neurons involved in the TCA cycle of glucose metabolism. Using oxaloacetic acid as raw materials, catalyzed by aminotransferase, aspartate is synthesized and stored in axon terminals. When nerve impulses are transmitted to axonal terminals, glutamate and aspartate are released by the presynaptic membrane and rapidly diffuse into the postsynaptic membrane; here, they bind to their corresponding receptors and prompt the opening of sodium and potassium channel gates to produce excitatory effects. The presynaptic membrane and glial cells reuptake a small amount of glutamate and aspartate.

Wistar rats were exposed to 30 mW/cm^2^ for 10 min of microwave radiation, and HPLC was used to detect changes in the levels of neurotransmitters, such as aspartate and glutamate, in the hippocampus 1, 7, 14, and 28 days after radiation. The results showed that the contents of aspartate and glutamate decreased 1 day after radiation, suggesting that acute EMR exposure could reduce the amount of excitatory amino acids in the hippocampus (17). Consistently, Ahmed et al. investigated the effect of EMR on the concentrations of amino acid neurotransmitters in the hippocampus, striatum, and hypothalamus of juvenile and young adult rats. The animals were divided into the control group and the exposure group, and the exposure group was subjected to 1,800MHz EMR, with SAR value of 0.843 W/kg, power density of 0.02 mW/cm^2^, 1 h daily for 1, 2, and 4 months. The results showed EMR induced significant decreases in glutamate and glutamine levels in hippocampal after 1 month (39). These data suggest that EMR can lead to a decrease in excitatory amino acid neurotransmitters in the hippocampus, which may affect the excitatory-inhibitory balance of neurons, thus causing a decline in learning and memory ability.

On the other hand, some studies have reported an increase in glutamate in the brain after radiation. Wang et al. exposed 160 Wistar rats to microwave radiation at 30 mW/cm^2^ for 5 min/day, 5 days/week, over a period of 2 months. The learning and memory ability, amino acid contents in the hippocampus and cerebrospinal fluid, and N-methyl D-aspartate receptor (NMDAR) subtype 2B (NR2B) expression were then investigated. Following microwave exposure, rats exhibited a significant decrease in learning and memory ability at 7 days and the glutamate contents in their hippocampus and cerebrospinal fluid increased, whereas the expression of NR2B protein decreased (20). Zhao et al. performed microwave exposure on 184 male Wistar rats for 6 min/day, over one month, at average power densities of 2.5, 5, and 10 mW/cm^2^. Morris water maze was applied to examine the learning and memory abilities. The concentrations of neurotransmitter in the hippocampus was detected by HPLC. The learning and memory ability of rats showed a significant decrease at 7, 14, and 1 month, following all three long-term microwave exposures. The concentrations of glutamate, aspartic acid, glycine, and GABA in the hippocampus were all increased for both 2.5 and 5 mW/cm^2^ groups, but these four amino acids were decreased in the 10 mW/cm^2^ group (40). These data further suggest the neurotransmitter disruption in the hippocampus might result in impairment of cognitive function caused by long-term microwave exposure.

Glutamate receptors are mainly constitutive of two types. The first type comprises ionic receptors, including NMDAR, kainate receptors (KARs) and α-amino-3-hydroxy-5-methyl-4-isoxazole receptors (AMPARs), which are conjugated with ion channels to form receptor channel complexes and mediate fast signal transmission. The second type encompasses metabolic receptors (mGluRs), which are conjugated to G proteins in the membrane. After being activated, these receptors act through a signal transduction system composed of a G-protein effector enzyme and a second messenger in the brain and produce a slow physiological response. Each NMDAR contains two binding recognition sites for glutamate and glycine, both of which are specific activators of the receptor (61). NMDARs are most often composed of two NR1 subunits and two NR2 subunits, and are highly permeable to Ca^2+^. NR1 is the basic subunit of NMDAR. For NR2 subunit, there are four subtypes including NR2A, NR2B, NR2C and NR2D. Glutamate binds to the NR2 subunits, while glycine binds to the NR1 subunit. The function of NMDARs is mainly dependent on the N-terminal domain of NR2 subunits (61, 62). Some studies have investigated the influence of EMRs on NMDAR expression in the brain.

Wang et al. exposed 220 male Wistar rats to microwave radiation, with frequency of 2.856 GHz, for 5 min/day, 5 days/week, over 6 weeks, at average power densities of 0, 2.5, 5, and 10 mW/cm^2^ respectively. For the 10 mW/cm^2^ group, the escape latency of rats significantly prolonged in the navigation tests of the Morris water maze, at 7 days, 1, 3, and 9 months after radiation. At 3 days after radiation, a significant impairment of rats in the probe trials was found in the 10 mW/cm^2^ group. Additionally, the protein levels of NR2A, NR2B and p-NR2B significantly decreased, and no significant change was observed for NR1 expression in the 10 mW/cm^2^ group from 1 day to 12 months after radiation. This suggests that decreases in NR2A, 2B and p-NR2B might contribute to the impairment of cognitive functions induced by microwave radiation (41).

Mausset et al. using a head-only exposure device in rats, found that a 15 min exposure to 900 MHz pulsed microwaves at a SAR value of 6 W/kg induced a strong glial reaction in the brain, a significant reduction in NR1 subunits in the cortex, a reduction in NR2A in the cortex and hippocampus, and a reduction in NR2B in the striatum. This suggests that exposure to high-power 900 MHz pulsed microwave radiation promotes specific NMDAR degradation processes (18). Moreover, Huang et al. exposed four-week-old female Wistar rats to 1800 MHz microwaves, at power densities of 0.5 mW/cm^2^ or 1.0 mW/cm^2^, for 21 days and 12 h each day. The expression of NR2A and NR2B in the hippocampal CA1, CA3 and dentate gyrus (DG) was determined by immunohistochemistry. For NR2A, the expression in the 0.5 mW/cm^2^ group was significantly lower than that in the 0 mW/cm^2^ group in CA3, but no significant changes were noted in CA1 and the DG. The expression in the 1.0 mW/cm^2^ group was significantly lower in CA1 and CA3, but no significant changes were found in the DG. For NR2B, the expression in the 0.5 mW/cm^2^ group was significantly lower than that in the 0 mW/cm^2^ group in CA1 and CA3. The expression in the 1.0 mW/cm^2^ group was significantly lower in CA1, CA3 and the DG (42). This further suggests that the decrease of NR2A and NR2B induced by microwave exposure depends on the dose of radiation and the district of hippocampus.

In addition, after microwave radiation exposure of 65 mW/cm^2^ for 20 min (SAR value 12.0 W/kg), the mRNA expression of the NR1 subunit in the hippocampus decreased at 3, 24 h, and 3 days, and the expression of the NR2A subunit decreased at 0 h, 3 h, and 12 h after microwave exposure. The mRNA expression of the NR2C subunit decreased at 0 and 24 h, but the expression of the NR2D subunit increased at 0, 12, 24 h, and 3 days after radiation. No significant changes in NR2B mRNA expression were observed (19). However, Xiong et al. exposed 48 male Wistar rats to 2.856 GHz, 30 mW/cm^2^ microwave radiation, for 10 min every other day three times. The mRNA expression of the NR2A subunit notably increased at 7 days, and the mRNA expression of the NR2B subunit in the rat hippocampus increased at 1 day after microwave exposure (21). Together, these results indicate that the composition of subunits comprising NMDARs can be altered and that the autoregulation of NMDARs can be destroyed in the rat hippocampus after exposure to microwave radiation. Furthermore, microwave radiation may affect the expression of excitatory amino acids.

### Effects of EMR on Inhibitory Amino Acid Neurotransmitters

GABA and glycine are the main inhibitory neurotransmitters in the brain, and GABA is an important neurotransmitter for approximately 50% of the synaptic sites in the central nervous system. GABA plays a critical role in the cerebral cortex, hippocampus, thalamus, basal ganglia and cerebellum, and has a regulatory role in various functions of the body, such as the regulation of emotion, memory and sleep, antihypertension, antifatigue, analgesia, etc. (63). GABA is produced in nerve endings catalyzed by glutamate decarboxylase. After release from the presynaptic membrane, most GABA diffuses to the postsynaptic membrane, causing an inhibitory effect in the postsynaptic membrane. The presynaptic membrane and glial cells reuptake a few GABA molecules, which are converted into succinic semi formaldehyde in mitochondria and then converted into succinic acid, which participates in the tricarboxylic acid cycle and provides a small part of the energy for glial cells and neural terminals (64, 65). Qiao et al. exposed Wistar rats to microwave radiation, with an average power density of 30 mW/cm^2^ for 5 min; then, HPLC was used to determine the GABA content released by hippocampal synaptosomes 6 h after exposure. The results showed that the amount of GABA released by hippocampal synaptosomes significantly decreased after radiation exposure (22). Zhang et al. investigated the effects of EMR exposure on the emotional behavior and spatial memory of adolescent male mice, with frequency of 1.8 GHz, and time duration of 4 weeks. The authors found that the levels of GABA and aspartic acid in the cortex and hippocampus significantly decreased after EMR exposure (66). These results suggest that EMR can reduce GABA neurotransmission.

Wang et al. exposed 80 Wistar rats to a 2.856 GHz pulsed microwave radiation, at a power density of 50 mW/cm^2^ for 6 min. The contents of amino acid neurotransmitters in the hippocampus were detected at 1, 3, 6, 9, 12, and 18 months after microwave exposure. The results showed that the glutamate to GABA ratio significantly decreased at 6 months after exposure (23). Noor et al. investigated the effect of 1 h of daily exposure to EMR, with a frequency of 900 Mz, SAR value 1.165 W/kg, power density 0.02 mW/cm^2^, on the levels of amino acid neurotransmitters in the midbrain, cerebellum, and medulla of adult male albino rats. The assessment of amino acid levels was applied after 1 h, 1, 2, and 4 months of radiation exposure. A significant glycine increase in the midbrain was observed after 1 month, followed by a significant increase in GABA after 4 months (9). These results further suggest that microwave radiation may affect the neuroregulatory function of GABA, resulting in an imbalance in excitation and inhibition in the central nervous system.

In the central nervous system, GABA acts as an inhibitory transmitter. GABA receptors include ligand-gated GABA (A) channels and G-protein-coupled GABA (B) receptors, which mediate inhibitory postsynaptic transmission throughout the nervous system (67). In one study, primary cultured rat cortical neurons were exposed to 900 MHz microwave radiation, with an average power density of 6 mW/cm^2^ and SAR value of 2.23 W/kg. As a result, the expression of neuronal GABA receptor proteins was significantly upregulated (24). Few studies have reported the effects of EMR on GABA receptors. Further investigation to clarify the role of GABA and its receptors during EMR exposure is necessary in the future. Overall, the above studies suggest that EMR can cause metabolic disorders of the inhibitory neurotransmitters GABA and glycine, which may lead to neuronal dysfunction by affecting the neuronal excitation-inhibition balance.

### Effects of EMR on Acetylcholine (Ach)

Cholinergic fiber projection from the basal forebrain to the cortex and hippocampus is the most important cholinergic system in the brain, and the cholinergic system plays a critical role in behavioral cognition. Ach is released from cholinergic nerve endings, and it was the first neurotransmitter to be measured in the brain. Changes in Ach in the extracellular fluid of the brain are closely related to functional changes in the central nervous system. Ach is synthesized by choline and acetyl-CoA under the catalysis of choline acetyltransferase (ChAT) and then taken up and stored by vesicles. When the neuronal presynaptic membrane is excited, Ach in synaptic vesicles is released into the synaptic cleft and acts on G-protein-coupled muscarinic acetylcholine receptors (mAChRs) or ligand-gated nicotinic acetylcholine receptors (nAChRs). Synaptic transmission efficacy can be changed by receptor-mediated membrane depolarization and downstream signal transduction, thus affecting learning and memory. Post-acting Ach is hydrolyzed to choline and acetic acid by acetylcholine esterase (AChE) and inactivated (68). The mode of action of Ach in learning and memory depends on the type of receptor it activates (69).

Few studies have been reported on the metabolism of Ach in brains exposed to EMR. Fujiwara et al. found that 2.45 GHz high-power microwave radiation caused transiently elevated Ach content in the mouse brain (25). Lai et al. found that acute exposure to 2.45 GHz, 0.6 W/kg microwave radiation for 20 min caused increased choline uptake activity in the frontal cortex, hippocampus, and hypothalamus of rats (26). Meanwhile, 2.45 GHz, 0.6 W/kg microwave radiation for 20 min/day for 10 consecutive days resulted in a decrease in the mAChR concentration in the rat frontal cortex and hippocampus, whereas radiation exposure of 45 min/day for 10 consecutive days resulted in an increase in the mAChR concentration in the rat hippocampus, both coinciding with a decrease in learning and memory ability. In addition, Krylova et al. found that 2.35 GHz, 1 mW/cm^2^ microwave radiation could induce a decrease in the functional activity of mAChRs in the rat cerebral cortex, though the number of mAChR receptors increase (27). We found an increase in Ach, ChAT and AChE in the rat hippocampus at 6 h and 3 days after microwave radiation, with a frequency of 2.856 GHz, and an average power density of 30 mW/cm^2^ for 15 min but no significant effect on the activity of ChAT and AChE. Moreover, we found that the expression of M1-, M3- and β2-type AChR mRNA was downregulated, whereas the expression of α4- and α7-type AChR mRNA was upregulated after radiation exposure. This indicates that the increased synthesis and metabolism of Ach and the disordered expression of Ach receptors may result in cholinergic system dysfunction and a decrease in cognitive function in the early period of acute microwave radiation exposure.

Furthermore, Testylier et al. found that the Ach released in the hippocampal CA1 area decreased after 1 h of microwave radiation exposure with 2.45 GHz and 4 mW/cm^2^, and the extracellular Ach concentration reached the lowest level of approximately 60% pre-exposure at 6 h after radiation (28). Other studies have shown that the M1 type of AChR is upregulated, the activity of AChE is increased, and the intracellular calcium concentration is increased in the hippocampus after long-term and low-dose microwave radiation at 2.45 GHz (44, 70). Derin et al. arranged Wistar rats into a sham-exposed group and 45 and 65 V/m exposed groups; the exposure group experienced 1 week of exposure at a frequency of 2.1 GHz. The protein and mRNA expression levels of AChE, ChAT, and VAChT in the hippocampus were examined using western blot and real-time PCR. The levels of AChE, ChAT, and VAChT were significantly lower in the rat hippocampus exposed to 65 V/m than in other regions (45). Additionally, sodium-dependent high-affinity choline uptake was measured in the striatum, frontal cortex, hippocampus, and hypothalamus of rats, after 45 min of short-term exposure to pulsed (2 μs, 500 pulses per second) or continuous 2,450 MHz microwaves in cylindrical waveguides. The average whole-body SAR value was 0.6 W/kg in all exposure conditions. The choline uptake was decreased in the frontal cortex after microwave exposure in all the radiation conditions (29). Gupta et al. reported a decrease in Ach content and an increase in AChE activity in the rat hippocampus caused by microwave radiation with 2.45 GHz, 1 h/day, for 28 consecutive days (46). Kunjilwar and Behari examined the effect of long-term exposure to RF-EMF on cholinergic systems in the developing rat brain, with a frequency of 147 MHz, subharmonics of 73.5 MHz and an amplitude of 36.75 MHz modulated at 16 and 76 Hz, 3 h/day, for 30–35 consecutive days. A significant decrease in AChE activity was found in exposed rats compared to control rats (71). These studies further suggested that disorders of Ach synthesis and metabolism are an important part of the cognitive dysfunction caused by EMR.

## Effects of EMR on Peptides and Other Neurotransmitters

Opioid peptides include β-endorphins, enkephalins and dynorphins, which are peptides with morphine-like activity in the brain. The Opioid receptors are G-protein-coupled receptors. Endogenous opioid receptors are able to inhibit adenosine cyclase, reduce voltage-dependent calcium channel currents or activate potassium channels, resulting in a decrease in membrane excitability and transmitter release, thus participating in the regulation of learning and memory processes (72). Lai et al. investigated subtypes of opioid receptors in the brain exposed to a 45 min of short-term exposure to pulsed microwaves (2,450 MHz, 1 mW/cm^2^, SAR value 0.6 W/kg) on cholinergic activity in the rat brain. The results showed that 3 opioid receptor subtypes blocked the decrease in cholinergic activity in the hippocampus induced by microwave radiation, suggesting that the opioid system is involved in microwave-induced hippocampal cholinergic activity decrease (30). There are few reports on the effect of EMR on peptide neurotransmitters. Lai et al. reported that after 45 min of exposure to pulsed 2,450 MHz microwaves (1 mW/cm^2^, SAR value 0.6 W/kg), rats showed learning impairment while performing in the radial arm maze to obtain food rewards. This indicated a deficit in spatial working memory function after EMR exposure. The microwave-induced learning deficit in the radial arm maze was blocked by pretreatment with the opiate antagonist naltrexone or a cholinergic agonist. This further suggests that both endogenous opioid neurotransmitter and cholinergic systems in the brain are involved in microwave-induced spatial memory deficits (31).

Nitric Oxide (NO) acts as a retrograde messenger in synaptic plasticity changes and long-term potentiation effects (48). Mice were exposed to computer electromagnetic radiation (30 x 10^14^-715 x 10^14^ Hz) with an intensity of 0.9 V/m (power density 0.22 μw/cm^2^) for either 6, 12, and 18 h/day for 30 continuous days. The results showed that the level of NO in the mouse brain gradually increased with prolonged radiation time (73). NO can pass through cell membranes by lipophilicity but is not released in the form of exocytosis; it acts through chemical reactions before becoming inactivated. In addition, NO can react with other free radicals and d-orbitals of transition metals. The most common for the latter is the interaction of NO with iron, because iron acts as a key component of abundant proteins, especially hemeproteins, involved in numerous physiological processes. Burlaka et al. exposed animals to ultrahigh frequency EMR of the non-thermal spectrum using the generator “Volna” (Ukraine) with impulse modulation and the following parameters: pulse duration 2 ms, pulse separation 10 ms, carrier frequency 0.465 GHz, and exposure duration 17.5 min. The energy flux density in the exposure area was 1.0–6.0 mW/cm^2^. Ultrahigh frequency EMR resulted in a significant increase in the level of NO synthesis in the mitochondria of neural cells in animal brain tissue and a significant increase in the activity of mitochondrial NO synthase (32). Considering the toxic effect of high NO concentrations on cells, the increase in NO may cause neuronal damage, which in turn leads to a decrease in learning and memory ability in mice.

## Possible Mechanisms Underlying Neurotransmitter Changes Caused by EMR

### Electrophysiological Changes

Neurophysiological mechanisms especially electrophysiological changes would lead to better understanding the neurotransmitter changes associated with EMR exposure. Several neuroimaging methods are used to illuminate the interference between brain electrical activity and EMR. For example, the changes of extracellular electrical potential in the cortex can be measured by EEG techniques, the regional changes of blood oxygen utilization can be detected with functional magnetic resonance imaging (fMRI) method during neuropsychological performance, and positron emission tomography (PET) reflects the cerebral metabolism (43, 74–76). Brain electrical activity originates from the fluctuation of membrane potential in the neuron. The transduction of a nerve impulse results in the postsynaptic potential and the following synaptic transmission, which could reflect the modulation of neurotransmission.

Many studies indicate an increase of cortical excitability and/or efficiency during EMR exposure, and this electrical activity changes may persist for several minutes post-exposure. In addition, an increase of cerebral metabolism (PET), a decrease of alpha activity, an increase of high beta and gamma frequency activity, increased reaction time, and disrupted sleep EEG were also induced by EMR exposure (77–82). Based on several methodologies, such as fMRI, PET, EMF-elicited event-related potentials (ERPs) (83, 84), and event-related desynchronization (ERD), and interhemispheric synchronization, frontal and temporal regions appear to be more susceptible (76, 81, 82, 85–87). In terms of the EMF-induced effects on cortical excitability and efficiency, several factors have been proposed, including alteration of dependent Na-K trans-membrane ionic channels, changes of cellular calcium homeostasis, increased cellular excitability, and modulation of cellular response to stress (86, 87). However, several inconsistent findings exist, and the heterogeneity of results may be due to methodological differences, statistical power, and interpretation criteria (88). Altogether, the abnormal brain electrical activity may reflect the modulation of neurotransmission induced by EMR, and result in the changes of neurotransmitters.

### Cell Membrane Damage

It is known that membrane is the first and an important target of EMF in cells. Cell membrane damage might result in neurotransmitter changes in the brain. Understanding the effects of EMR on neurotransmitters is critical for further determining the targets of EMR in cells. EMR can alter cell membrane permeability such as changes in calcium, ionic distribution and ion permeability (89). Calcium is one of the important signaling substances, and an imbalance of calcium homeostasis can alter many functions of the cell. Previous studies have showed that EMR exposure can alter the calcium channels and receptors on the cell membrane, and influence transport of calcium ions over the cell membrane, which play an important role in cell signaling pathways, and in turn may affect the response of neurotransmitters (90, 91). It was reported that the number of opened calcium channel increased with the presence of EMFs, which might resulting in the increased intracellular calcium concentration under EMR exposure (92). In addition, the changes of intracellular calcium levels can trigger unusual synaptic action or cause neuronal apoptosis. This in turn can exert an influence on the neurotransmission of learning and memory process (93).

Additionally, the enhanced activity of voltage-gated calcium channels (VGCCs) have been delineated, after exposure to EMR in many cell types (94–96). Previous studies used the activity of VGCCs as an indicator of microwave radiation induced changes in ion channels (96, 97). The level of neurotransmitters can indicate the membrane properties, such as the expression level of synaptic vesicular-associated proteins, can indicate the function of the synaptic vesicular membrane (22, 98). It was reported that, EMR activation of VGCCs causes a rapid increase in intracellular calcium, nitric oxide, and peroxynitrite (99). However, a recent study on the effects of 2.856 GHz pulsed microwave radiation in the primary hippocampal neurons, reported that the total cellular calcium, the levels of calcium in endoplasmic reticulum and mitochondria all decreased after microwave exposure, suggesting calcium efflux during microwave radiation (100). Although many animal studies have suggested about the effects of EMR on the calcium efflux and influx in the neurons (101–103), the results regarding the effects of EMR on the membrane integrity and permeability are still unclear. The changes of membrane permeability may result in the damage of membrane integrity, and lead to the changes in brain neurotransmitter imbalance. In this regard, further studies by various duration and dose of EMR are needed to investigate the effects of EMR on the relationship of neurotransmitters and cell membrane permeability.

### Abnormal Signal Transduction

It is known that neurotransmitter and its receptors are involved in various signaling related to cell proliferation, apoptosis, differentiation and inflammation. The crosstalk between neurotransmission and cell signaling may in turn affect the metabolism and transport of neurotransmitters. EMR exposures produce the main pathophysiological effects via excessive calcium signaling and the peroxynitrite pathway, and the diverse non-thermal effects of EMR are produced via VGCC activation (104). As the energy source of the cell, the mitochondrial calcium reaction was influenced by the alterations in calcium signaling pathways in response to the effects of EMR exposure (90). In addition to calcium signaling changes, EMR can cause activation of free radical processes and overproduction of reactive oxygen species (ROS) in neurons (53, 104–108). Due to the dependent on oxidative phosphorylation for energy, neurons are vulnerable for oxidative stress compared to other cells. During EMR exposure, the occurrence of oxidant-antioxidant imbalance in the brain leads to oxidative stress (109). Both NO and superoxide (${\text{O}}_{2}^{-}$) are elevated by increased calcium, resulting in the increase of peroxynitrite (ONOO^−^) levels. The various oxidants act to produce greatly elevated NF-kappa B (NF-κB) activity, leading to inflammation (110). In addition, NF-κB signaling is reported to be involved in neural immune response, synaptic plasticity, learning and memory, neuroprotection and neurodegeneration (111, 112). It has been shown that EMR exposure leads to up-regulated elements belonging to apoptotic pathways, which results in neuronal apoptosis (113, 114). The probable mechanisms are mainly attributed to increased ROS generation following EMR exposure.

The energy of non-ionizing radiation is not enough to directly break chemical bonds, and therefore the occurrence of DNA damage with non-ionizing EMR exposures is primarily a consequence of generation of ROS, followed by oxidative stress. Numerous animal experiments have clearly demonstrated that non-thermal EMR can cause oxidative stress (115, 116), particularly in the brain (3, 117–119). It has been documented that non-thermal EMR exposure of 900 MHz or 2.45 GHz in rats, either short-term or long-term, can trigger neuronal dysfunction and apoptosis of hippocampal pyramidal cells (117, 120) and cerebellum Purkinje cells (121) through induction of oxidative stress. In addition, the mitogen-activated phosphokinase (MAPK) pathway plays a key role in cell proliferation and metabolism. The phosphorylation of transcription factors in the downstream occurs after activation of the MAPK cascades pathway (89, 122). The proliferation and survival of different cell types can be stimulated by low concentrations of free radicals. The effects of ROS on cell proliferation, is an important secondary messenger in the physiological process, and ROS plays a key role in the regulation of cytosolic calcium homeostasis. The protein phosphorylation and activation of the AP-1 family factors and nuclear factor kappa B (NF-κB) is regulated by the level of cytosolic calcium (123). Activation of the protein kinases pathways regulates the physiological response to EMR exposure including neurotransmitter imbalance, but the detailed mechanisms are still unclear.

## Discussion

According to the time duration of EMR exposure, we divided all the references including neurotransmitter measurement in the brain into two groups: short-term (within one week) exposure and long-term (more than one week) exposure groups. It is apparent from the listed reference in Tables 1, 2 that, no obvious difference was observed for neurotransmitter changes between the short-term (Table 1) and long-term (Table 2) EMR exposure. It is known that the response to non-thermal EMR depends on both power density and duration of exposure. Some studies show no effect under fixed short-term EMR exposures, but this does not imply no effects under longer-term exposures (5, 124). In a recent review, Leach et al. analyzed 2,653 papers captured in the database examine the bioeffect outcomes in the 300 MHz−3 GHz range. The results showed three times more biological “Effect” than “No Effect” papers (125). Although some studies report no effect on the tested indicators, there are studies find the significant effect in many cases. This inconsistency might due to the lack of replication between studies. It is challenging for undertaking a literature review or comparing findings between relevant scientific papers, due to the subject, various experimental methodology and changing exposure parameters in the available studies. Notwithstanding animal models can only provide a strong indication of risks to humans, and the exchange formula or conversion rules between animal studies and human biological effects are far from clarified. The development of reliable safety standards has analyzed such parameters as power density, dose, and duration of exposure, and this would protect against the detrimental health effects of EMR exposure at non-thermal intensities.

**Figure 1:**
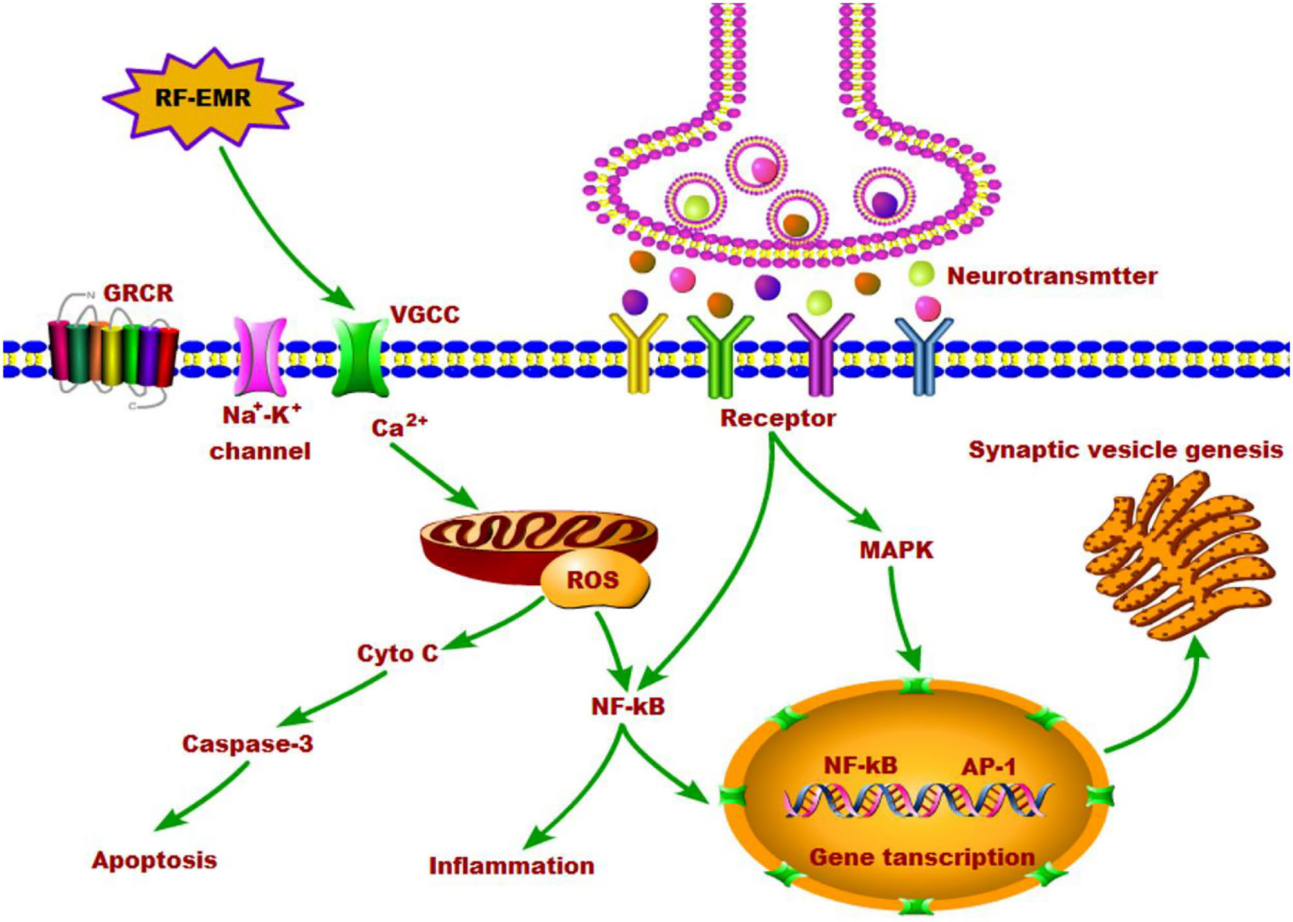
The effects of RF-EMR exposure on neurotransmitters in the brain and the possible underlying mechanisms.

Many evidences indicate that EMR alter several aspects of calcium function in cells. In spite of numerous studies reporting altered calcium metabolism upon exposure to radiofrequency electromagnetic fields, the underlying mechanisms of these effects are still not clear. However, some studies have suggested that the calcium activation could be the initial event leading to alteration in protein configuration, followed by generation of ROS and ultimately activation of the molecular apoptosis pathways (101). Lushchak et al. reported that EMR exposure may firstly produce the free radicals in the brain and later they are converted to ROS (126). The elevation of ROS level can attack various biomolecules in the cell. The raised ROS can also in turn trigger calcium release, and then activate the genetic factors leading to DNA damage (110). Any alteration in gene and enzyme levels, may result in the activation of downstream signaling (114), particularly the mitochondria-dependent caspase-3 pathway can cause the apoptosis of neurons (113, 127), which would lead to altered behavioral manifestations and pathophysiological changes in the brain. In a word, EMR exposure does increases the intracellular calcium and the formation of ROS, which would alter the cellular function eventually and lead to numerous biological effects including neurotransmitter imbalance. We summarized the effects of EMR on neurotransmitters in the brain and the possible underlying mechanisms in Figure 1.

Though we narrow down to biochemical imbalance to simplify explanation for changes of each neurotransmitter, the combined effects of neurotransmitters still deserves attention. It is also possible that the various neurotransmission effects following EMR exposure in animals might be due to combined effects in various brain regions, such as neurophysiological changes, increase of calcium and ROS, and thereby cell membrane damage and the downstream signaling changes. An imbalance in the excitation-inhibition imbalance of neurons resulting from neurotransmitter changes, would alter behavior, and it might do so without evident structural changes. Currently, the neurochemical mechanisms of EMR exposure are still unclear. Further study in this regard is needed and will reveal much clearer picture of brain mechanisms caused by EMR.

## Conclusion

In summary, research on the synthesis, metabolism and transport of neurotransmitters in the brain by EMR is increasing gradually, but due to the different parameters of EMR, experimental objects and conditions, the experimental results are not very consistent and comparative. Therefore, the effects of EMR on the metabolism and transport of neurotransmitters have not been clarified. Moreover, the role of neurotransmitters and their mechanism in the neurobehavioral dysfunction induced by EMR have not been revealed. Further detailed studies are needed. On the other hand, because of the complex diversity of neurotransmitters in the brain, the interaction, cotransmission and coregulation of neurotransmitters make it difficult to distinguish the primary and secondary changes of each neurotransmitter. Furthermore, the interaction of different neural nuclei in the brain constitutes sophisticated neural circuits, which is the fundamental basis of how the brain performs functions. Consequently, the regulation of neural circuits may be involved in the neurotransmitter disorder of the brain induced by EMR.

## Future Perspectives

Recently, novel techniques in brain science, such as neuroviral tracers, neuroimaging and neuroelectrophysiology, have been rapidly developed. These techniques were devised especially for the development and wide application of brain intervention techniques, including optogenetics and chemical genetics. Moreover, these advances have provided new methods to study the neurobiological effects of EMR at the neural circuit level. Notably, the G-protein-coupled receptor activation-based (GRAB) sensor can directly measure neurotransmitter release and monitor the activity of neurotransmission *in vivo* (128). Combined with fiber photometry recording, the GRAB sensor enables sensitive detection of single-trial neurotransmitter dynamics in multiple brain regions in mice performing a variety of behaviors (82). With these new techniques in neuroscience, studying the effects of EMR on neurotransmitter metabolism and the transport of neurotransmitters at the neural circuit level is expected to overcome the challenges inherent in investigating the neurobiological effect of EMR and its mechanisms and open novel pathways for the exploration of preventive targets and interventions.

## Author Contributions

CH wrote the paper and outlined this manuscript. HZ and YL provided a detailed guidance throughout the article. All the authors read and approved the final manuscript.

## Conflict of Interest

The authors declare that the research was conducted in the absence of any commercial or financial relationships that could be construed as a potential conflict of interest.

## Publisher's Note

All claims expressed in this article are solely those of the authors and do not necessarily represent those of their affiliated organizations, or those of the publisher, the editors and the reviewers. Any product that may be evaluated in this article, or claim that may be made by its manufacturer, is not guaranteed or endorsed by the publisher.
